# High-Quality Spherical Silver Alloy Powder for Laser Powder Bed Fusion Using Plasma Rotating Electrode Process

**DOI:** 10.3390/mi15030396

**Published:** 2024-03-14

**Authors:** Hao Li, Shenghuan Zhang, Qiaoyu Chen, Zhaoyang Du, Xingyu Chen, Xiaodan Chen, Shiyi Zhou, Shuwen Mei, Linda Ke, Qinglei Sun, Zuowei Yin, Jie Yin, Zheng Li

**Affiliations:** 1 Gemmological Institute, China University of Geosciences, Wuhan 430074, China; haoli@cug.edu.cn (H.L.); 20121001484@cug.edu.cn (Q.C.); zhaoyang_du@cug.edu.cn (Z.D.); 945365445@cug.edu.cn (X.C.); 17719396590@cug.edu.cn (X.C.); zhoushiyi@cug.edu.cn (S.Z.); sunqinglei@cug.edu.cn (Q.S.); yinzuowei1025@163.com (Z.Y.); yinjie@cug.edu.cn (J.Y.); 2 Hubei Engineering Research Centre of Jewellery, Wuhan 430074, China; 3 Key Laboratory of Superlight Materials and Surface Technology, Ministry of Education, College of Materials Science and Chemical Engineering, Harbin Engineering University, Harbin 150001, China; zhangshenghuan15@163.com; 4 Nantong Jinyuan Intelligence Manufacturing Technology Co., Ltd., Nantong 226010, China; meishuwen@jyznjs.com; 5 Shanghai Engineering Technology Research Centre of Near-Net-Shape Forming for Metallic Materials, Shanghai Spaceflight Precision Machinery Institute, Shanghai 201600, China; kelinda_casc@163.com

**Keywords:** Ag alloy, plasma rotating electrode process, continuous casting, laser powder bed fusion, hardness

## Abstract

The plasma rotating electrode process (PREP) is an ideal method for the preparation of metal powders such as nickel-based, titanium-based, and iron-based alloys due to its low material loss and good degree of sphericity. However, the preparation of silver alloy powder by PREP remains challenging. The low hardness of the mould casting silver alloy leads to the bending of the electrode rod when subjected to high-speed rotation during PREP. The mould casting silver electrode rod can only be used in low-speed rotation, which has a negative effect on particle refinement. This study employed continuous casting (CC) to improve the surface hardness of S800 Ag (30.30% higher than mould casting), which enables a high rotation speed of up to 37,000 revolutions per minute, and silver alloy powder with an average sphericity of 0.98 (5.56% higher than gas atomisation) and a sphericity ratio of 97.67% (36.28% higher than gas atomisation) has been successfully prepared. The dense S800 Ag was successfully fabricated by laser powder bed fusion (LPBF), which proved the feasibility of preparing high-quality powder by the “CC + PREP” method. The samples fabricated by LPBF have a Vickers hardness of up to 271.20 HV (3.66 times that of mould casting), leading to a notable enhancement in the strength of S800 Ag. In comparison to GA, the S800 Ag powder prepared by “CC + PREP” exhibits greater sphericity, a higher sphericity ratio and less satellite powder, which lays the foundation for dense LPBF S800 Ag fabrication.

## 1. Introduction

Silver (Ag) and silver alloys have high thermal conductivity, electrical conductivity and antibacterial properties with broad application prospects in various fields such as aerospace, medical, jewellery and electric devices. Silver alloy with 80% silver content (S800 Ag) has high toughness and oxidation resistance whilst retaining the ductility of pure silver and is widely used in the manufacture of large silverware and silver jewellery [1,2,3,4,5,6]. However, the limitations of the traditional casting process in terms of the freedom to shape the part limits the designer’s creative freedom and the range of applications for the material. Laser powder bed fusion (LPBF) is a metal additive manufacturing technology that melts discrete powders materials and deposits them layer by layer [7,8,9,10,11,12,13]. The layer-by-layer forming feature of the powder bed enables the manufacture of parts with complex shapes [14], expanding the freedom of part design [15,16,17,18,19]. The utilisation of silver alloy materials, which possess excellent properties, combined with the shaping benefits of LPBF, will expand the range of applications for silver alloys in smart electronic products, wearable devices and medical devices.

In recent years, studies on the additive manufacturing of silver and silver alloys have mainly focused on the LPBF forming process [20,21,22], microstructure, performance [23,24,25,26] and microscopic defects [27], but few studies have been carried out on powder’s preparation. Gas atomisation (GA), the plasma rotating electrode process (PREP) and plasma atomisation (PA) are the three main methods for the preparation of LPBF metal powders [28]. In particular, the GA and PREP methods have been widely used in the industrial production of various alloy powders such as nickel-based, titanium-based and iron-based alloys [29]. The use of wire as a raw material in PA results in lesser productivity and higher costs compared to the previous two methods [30]. Currently, the silver-based powders used in LPBF are mainly prepared by GA. The powders prepared by GA have about 40% non-spherical particles and satellite particles [31,32], which affects the flowability, adhesion and filling properties of the powders. These powders particles are considered to be a major obstacle to the formation of a uniform and dense powders layer in the process of powders recoating [29]. Gao et al. compared non-spherical and spherical particles at a layer thickness of 80 μm by simulation and found that non-spherical particles reduce the packing density of the powder and increase the surface roughness of the powder bed, and the elongated gaps formed among the joined particles lead to severe breakups of the liquid metal, contributing to the destabilisation of the melt pool [32]. Chu et al. conducted a comparison of the forming effect of LPBF with and without satellite particles. They found that the porosity of the formed sample with satellite particles (0.2~1.7%) was consistently larger than that of the formed sample without satellite particles (0.1~0.2%). Satellite particles affect the uniformity of powder deposition, resulting in the formation of unfused or unfilled defects within the parts [33].

The plasma rotating electrode process (PREP) is a suitable method for producing metal powders used in additive manufacturing. PREP powders have the advantages of good sphericity, fewer satellite powders and narrow particle size distribution [34,35], which is superior to GA powders. However, there is no relevant research on the preparation of PREP powders for silver-based materials.

The conventional silver alloy used in mould casting is constrained by its material and process, resulting in low hardness, poor density and the inadequate uniformity of the crystalline structure. Additionally, the casting electrode rod is prone to defects such as air holes, looseness and shrinkage holes, making it unable to withstand the high-speed rotation during the PREP process. The mould casting silver electrode rod can only be used in low-speed rotation, which negatively affects particle refinement.

This research addresses the issues of low hardness and numerous defects in the traditional mould-casting of silver alloy, which render it unsuitable for PREP powder production. This study provides a new way to enhance the hardness of the silver alloy electrode rod through continuous casting, which allows it to be used in PREP with high-speed rotation. PREP silver alloy powders with high sphericity and a high sphericity ratio have been successfully prepared. Finally, dense silver alloy samples have been prepared using LPBF, which verified the high quality of the PREP powders.

## 2. Materials and Methods

### 2.1. Materials

Pure silver ingots and copper alloy particles (Noble metal of Huanggang Co., Ltd., Huanggang, China) were used as raw materials; silver alloy rods of φ30 mm were prepared by continuous casting for powder preparation.

### 2.2. Preparation of Silver Alloy Rod

**Figure 1:**
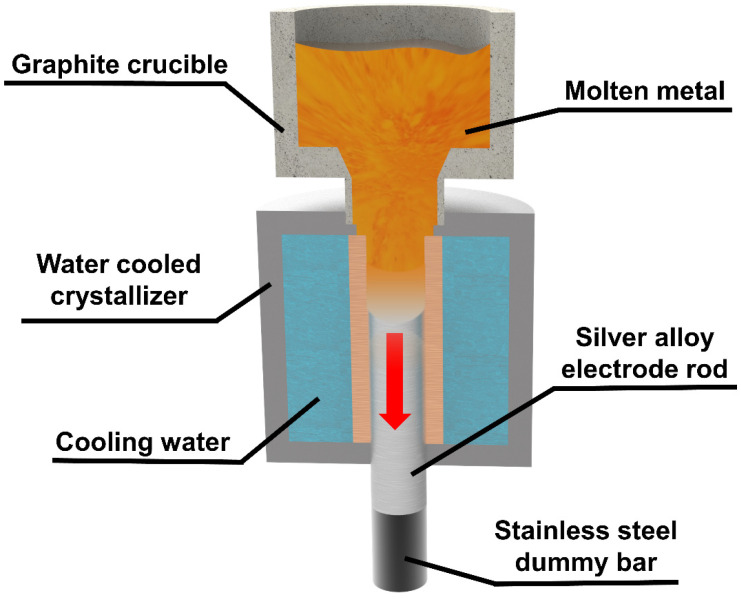
Schematic diagram of the principle of continuous casting.

Continuous casting is a technology in which melted metal is continuously poured into a water-cooled crystalliser and the solidified casting is continuously pulled out from the other end of the crystalliser [36,37], as shown in Figure 1. The quickly cooled metal has a uniform structure with high density. It has better mechanical properties and higher hardness, which provides support for stable high-speed rotation during subsequent PREP.

In this study, φ30 mm rods were prepared by continuous casting, and the casting was carried out under nitrogen environment. The ceramic crucible of the continuous casting machine was preheated to 1000 °C, and pure silver ingots and copper alloy particles were weighed into the graphite crucible according to the ratio (Ag:Cu alloy = 80:20) until complete melting. Then, the cooling water was turned on and the pressure was set to 0.15 MPa. The solidified silver alloy was pulled out by stainless steel dummy bar, and pulling speed was set to 30 mm/min. A silver alloy electrode rod was formed.

### 2.3. PREP Preparation of Silver Alloy Powders

**Figure 2:**
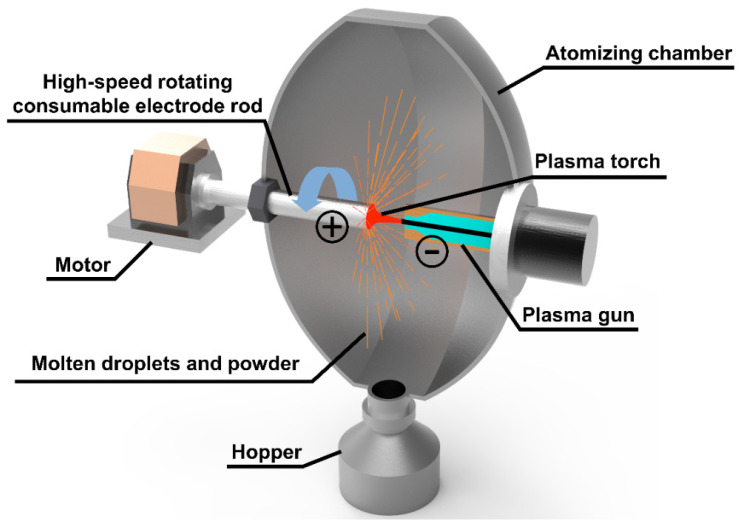
PREP principal diagram.

Sailong Additive Technology Co., Ltd., (Xi’an, China) of SLPA-D desktop PREP system was selected to prepare the S800 Ag powder (Figure 2). In this system, the electrode rod is inserted into the atomisation chamber through the mechanical shaft, and the electrode is driven to rotate at high speed; the electrode rod work as the anode in the powder preparation process, and the plasma gun in the atomisation chamber forms a conduction circuit, generating high-temperature plasma torch. The end face of the rods rotating at high speed is melted, crushed and condensed by the high-temperature plasma torch to form powders [38]. The parameters of PREP silver alloy powder preparation obtained through research and exploration are shown in Table 1. The powder preparation process was carried out in argon gas. The electrode rotation speed was 25,000–37,000 rpm, the DC current was 500–700 A and the feed rate was 3.5–4.5 mm/s.

### 2.4. Preparation of Samples for Laser Powder Bed Fusion

The SISMA MYSINT100 platform was employed to LPBF fabrication, which was equipped with a Nd: YAG fibre laser with a wavelength of 1064 nm, a laser power of up to 200 W and a spot diameter of 30 μm. All samples were constructed in an argon atmosphere (with a residual oxygen content of 0.5 vol%).

**Figure 3:**
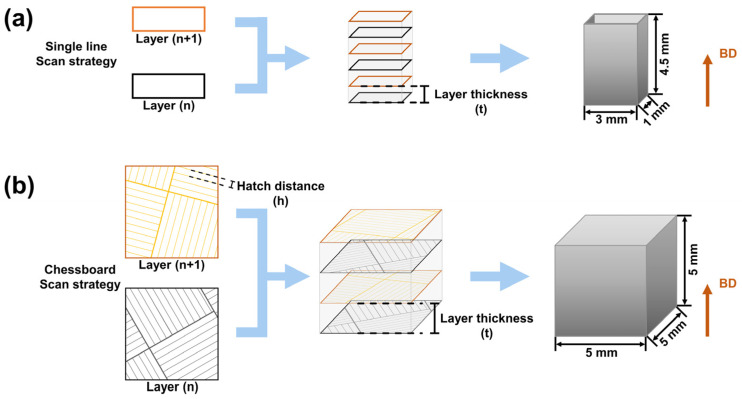
Sample manufacturing strategy: (**a**) single-track scanning strategy; (**b**) checkerboard scanning strategy.

The process parameters and scanning strategy were designed by the Materialise AutoFab mysint 2.0 (b424336) software. Two sets of samples were used for forming, as shown in Figure 3. The single-track sample (Figure 3a) was constructed using a single-track scanning strategy to profile the outer edge of the rectangular body, which was used to simulate the actual melt pool width in order to calculate the hatch distance of bulk sample (Figure 3b). The bulk sample was scanned using a checkerboard scanning strategy (layer thickness of 40 μm), with each layer divided into 4 × 4 mm square bulks, each layer (n + 1) rotated by 45° with respect to the previous layer (n) and each bulk translated by 4 mm along the positive direction of the X-axis and the positive direction of the Y-axis [39,40,41], as shown in Figure 3b.

### 2.5. Measurement of Microstructure and Physical Property

The morphology of the silver alloy powders (acceleration voltage 20 kV) and the grain structure of the silver alloy rod (acceleration voltage 15 kV) were determined by scanning electron microscope (TESCAN MIRA LMS, TESCAN Ltd., Brno, Czech Republic). The particle size distribution statistics of the powders were analysed by Mastersizer 3000 (Malvern Panalytical Ltd., Malvern, UK) laser diffraction particle size analyser. The laser absorptivity of the powders was analysed by JASCO MSV 520 UV-Vis/NIR Micro Spectrophotometers (JASCO International Co., Ltd., Tokyo, Japan).

Image-Pro Plus was used to calculate the sphericity and sphericity ratio of the powders. The sphericity was calculated according to the roundness of the powder particles in the SEM image (Formula (1)). *S* is the area of the powder particles, and *C* is the circumference of the powder particles; we measured the long axis and short axis of the powder particles. Particles with a ratio of the long and short axes ≤ 1.2 were considered spherical. Sphericity ratio was calculated by dividing the number of spherical particles by the total number of samples [42].

(1) $$\mathrm{Sphericity}=\frac{4\pi \times S}{C^{2}}$$

Silver alloy rods and LPBF samples were polished and etched (50 mL ammonia water + 100 mL H_2_O_2_ (3 vol%) + 50 mL distilled water) for microstructure analysis. The surface of the sample was observed with an optical metallographic microscope (Nikon ECLIPSE MA100N, Nikon Corp., Tokyo, Japan) to characterise the sample defects, microstructure and morphology. The density of LPBF samples were measured by Archimedes’ method. The hardness of silver alloy rods and LPBF samples (constant load 1 N, dwell time 10 s) were obtained using Wilson VH1102 Vickers hardness tester (Buehler Ltd., Lake Bluff, IL, USA). The NETZSCH LFA 467 (Netzsch, Bavaria, Germany) laser thermal conductivity meter was used to measure the thermal conductivity of LPBF samples.

## 3. Results and Discussion

### 3.1. Continuous Casting of Silver Alloy Rods

#### 3.1.1. Microstructure of Silver Alloy Rods

**Figure 4:**
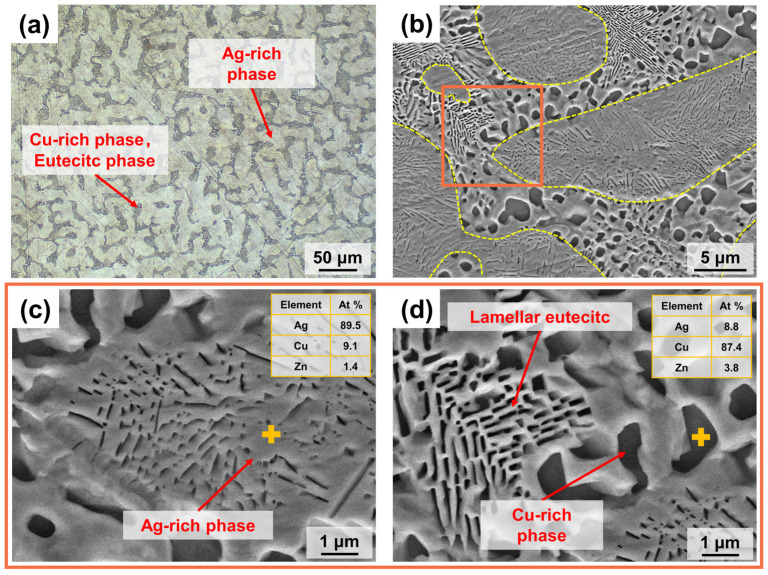
Microstructure of S800 Ag rod cross section; (**a**) Optical microstructure; (**b**) SEM microstructure, α − Ag phase was marked by yellow dashed box; Area of EDS analysis was marked by orange box (**c**) EDS point analysis of α − Ag phase; (**d**) EDS point analysis of eutectic phase and β − Cu phase. The EDS point was marked by yellow +.

Figure 4a displays the optical micro-metallographic organisation of the cross-section of the S800 Ag rod. The image reveals a fine dendritic structure, consisting of three phases: the primary phase (α − Ag), the degenerated eutectic copper (β − Cu) and the eutectic phase (α + β) [43]. The dendrites in the light-coloured area are the primary phase (α − Ag), while the darker region between the primary phases contains the eutectic phase (α + β) and the degenerated eutectic copper (β − Cu).

Cu-rich nanosized particles and continuous lamellar structures were observed inside the α − Ag phase, and EDS analysis showed that the Cu content of the α − Ag phase was 9.1 At% (Figure 4c). During the process of cooling and solidification, the primary phase (α − Ag) grows first in the form of dendrites and Cu is enriched at the edge of head area of the dendrites, resulting in the formation of the eutectic and β − Cu phases. In Figure 4b, the yellow dotted line represents the boundary of the α − Ag phase. At the edge of the α − Ag phase, there are noticeable large black phases formed by aggregates of the β − Cu solid solution with a copper content of 87.4 At% (Figure 4d). The eutectic phase (α + β) is present in a stratified eutectic structure located between the dendrites of the α − Ag phase (Figure 4d).

#### 3.1.2. Vickers Hardness of Silver Alloy Rod

**Figure 5:**
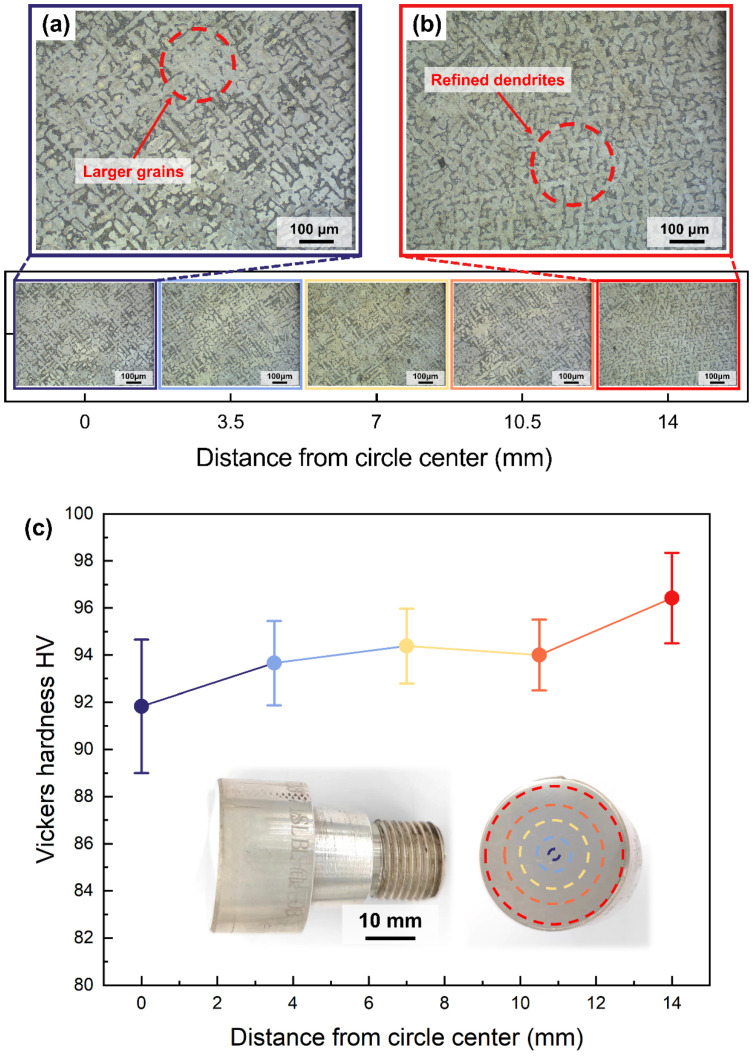
Vickers hardness and microstructure of continuously cast S800 Ag rods at different distances from the centre of the circle, (**a**) microstructure of the central region of the rod, (**b**) microstructure of the edge region of the rod. (**c**) Vickers hardness of continuously cast electrode rod at different distances from the centre of the circle, the test points are marked by dashed circle on the cross-section of the continuously cast rod.

Figure 5 shows the micro-metallographic structure of the S800 Ag electrode rod cross-section at various positions ranging from the centre to the edges. The grain size of the centre region (Figure 5a) is bigger than that of edge region (Figure 5b) due to the water cooling during continuous casting process. The water-cooled crystalliser plays a crucial role in rapidly cooling the outer edge, thereby contributing to grain refinement, which significantly enhances the hardness of silver alloys.

The hardness of the S800 Ag electrode rod was measured at different locations of the cross-section from the centre to the edge in Figure 5c. The hardness of the electrode rod increases as the distance from the centre of the circle increases. The average hardness at the centre is 91.82 HV, while at the outer edges, it is 96.42 HV, with a maximum of 98.90 HV. These findings align with the microstructure above, which shows that the surface hardness of the electrode rod is enhanced by fine grain reinforcement [37] using a water-cooled crystalliser. The traditional mould casting of S800 Ag results in a hardness of 74 HV [44]. The hardness of S800 Ag in this study is 24.08% higher in the centre region and 30.30% higher in the outside edge region compared to mould casting. It was proven that continuous casting provides an effective way to improve the hardness of silver alloys. The improvement in surface hardness is beneficial in preventing the rod from deflecting and bending during high-speed rotation. This ensures stable high-speed rotation during the following PREP powder preparation process.

### 3.2. Physical Properties of Silver Alloy Powders

**Figure 6:**
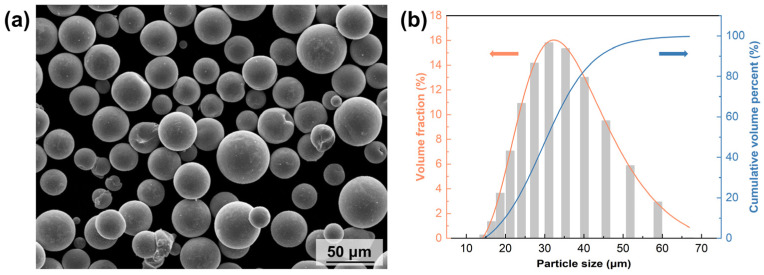
SEM image and particle size distribution of silver alloy powders: (**a**) SEM image of S800 Ag powders, (**b**) particle size distribution of S800 Ag powders.

Figure 6 shows the SEM morphology and particle size distribution of the S800 Ag powders prepared by PREP. The corresponding data for the powders can be found in Table 2. The particle size of the powders is mostly in the range of 15–60 μm, which is in line with the requirements of LPBF printing. The primary constituents of S800 Ag powders consist of 78.8 wt.% Ag, 18.5 wt.% Cu and 2.7 wt.% Zn, and the powder laser absorptivity (wavelength: 1064 nm) is 64.60%.

**Figure 7:**
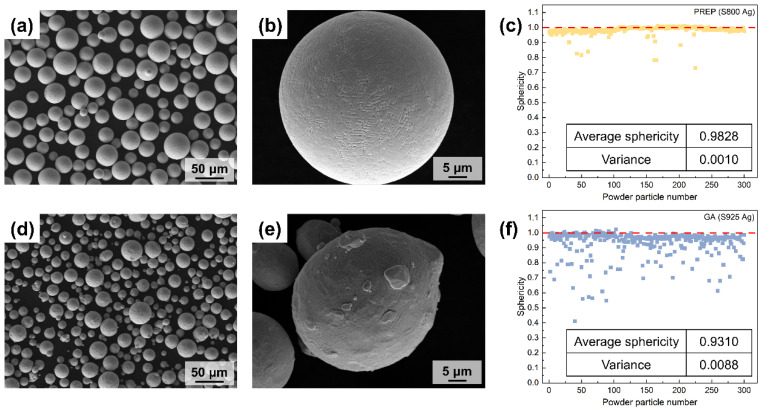
SEM morphology and powders sphericity of PREP silver alloy powders and GA silver alloy powders; (**a**,**b**) PREP S800 Ag powders morphology; (**c**) PREP S800 Ag powders sphericity, red dotted line is the sphericity value corresponding to perfect spherical; (**d**,**e**) GA S925 Ag powders morphology; (**f**) GA S925 Ag powders sphericity, red dotted line is the sphericity value corresponding to perfect spherical.

Figure 7 shows the structure of silver alloy powders (S800 Ag) prepared using PREP and silver alloy powders (S925 Ag) prepared using gas atomisation (GA), provided by Legor Group. The majority of powders prepared by PREP show a high degree of sphericity, and satellite powders are rarely observed. Fine dendrites can be observed on the surface of the powders (Figure 7b), which is due to the rapid cooling of droplets by centrifugal rate and thermal exchange with the atmosphere during the PREP. The molten metal liquid film breaks up due to centrifugal force, resulting in the formation of small droplets. These little liquid particles have thermal exchange with the protective atmosphere, swiftly cooling to form fine dendrites. Compared to PREP powders, GA powders are less spherical (Figure 7d) and have more satellite particles, most of which have irregular small particles adhering to the surface of the powders (Figure 7e), seriously affecting the sphericity of the powders.

The sphericity of the PREP and GA silver alloy powders was calculated by computationally analysing the roundness of the particles (Figure 7c,f). A sphericity value of 1 indicates that the particles are perfectly spherical, and the closer the sphericity value is to 1.00, the better the sphericity of the powders. The majority of the PREP silver alloy powders have a sphericity near to 1 and only a small number of powders particles have a sphericity lower than 0.90. The average sphericity is 0.98, which indicates that the preparation of PREP powders has good sphericity. The few particles with sphericity lower than 0.9 are mostly satellite powders and long stripes of powders. Figure 7f shows the sphericity of the GA silver alloy powders. The figure reveals that there are numerous powders with sphericity values below 0.90. The average sphericity of these powders is 0.93, which is lower than that of PREP. This finding aligns with the observations obtained from the SEM images, which indicate that GA preparation results in more irregular and satellite powders. Additionally, the variance calculation of the sphericity values further confirms that the distribution of sphericity in GA powders is more dispersed and heterogeneous compared to PREP powders.

**Figure 8:**
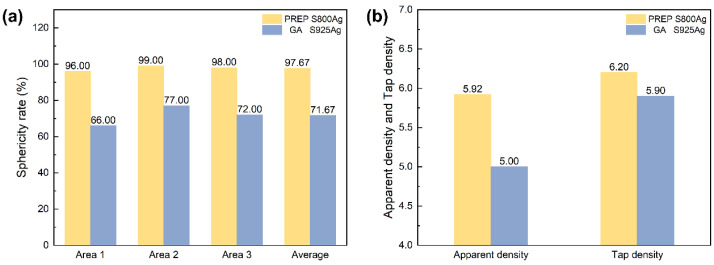
(**a**) sphericity ratio of PREP S800 Ag and GA S925 Ag; (**b**) apparent density and tap density of PREP S800 Ag and GA S925 Ag.

The sphericity ratio calculation findings for silver alloy powders generated using PREP and GA are displayed in Figure 8a. The sphericity ratio refers to the proportion of spherical particles (the ratio of the long and short axes of the particle image ≤ 1.2) in the whole sample. Only 71.67% of GA silver alloy powders satisfied the spherical requirement. In contrast, the silver alloy powders prepared by PREP in this study had a sphericity ratio of 97.67%, which resulted in superior apparent density and tap density compared to the GA powders (Figure 8b). The presence of non-spherical particles and satellite particles in the GA powders resulted in numerous gaps in the powder packing, leading to decreased apparent density and tap density. In conclusion, the silver alloy powders prepared by PREP were significantly better than those prepared by GA in terms of the sphericity, sphericity ratio, apparent density and tap density.

### 3.3. Optimisation of LPBF Process

#### 3.3.1. Single-Track Samples

**Figure 9:**
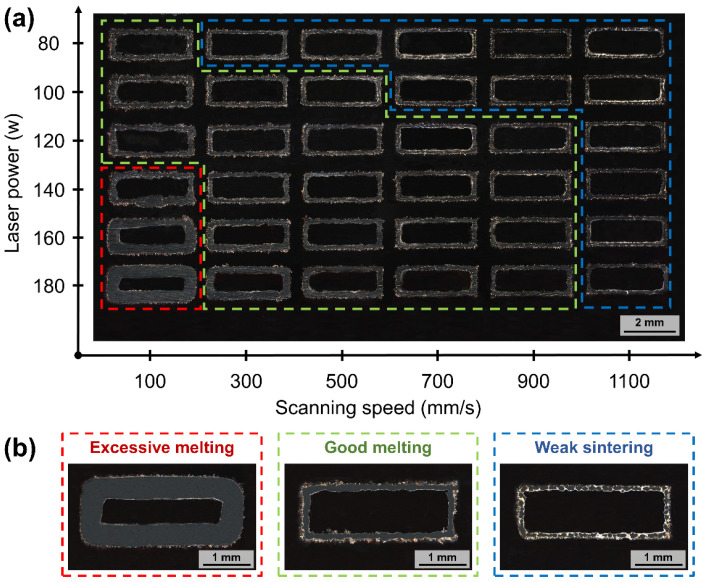
Process parameters of S800 Ag single-track morphology and processing window schematics, (**a**) cross-sectional morphology of single-track samples at different process parameters; (**b**) three processing windows of single-track schematics.

The cross-section of the S800 Ag single-track sample is shown in Figure 9. The single line width is employed to simulate the width of the melt pool, which is used to control the forming parameters of the bulk. The processing window has been divided into three regions based on the breadth and continuity of the melt pool (see the domain Figure 9b), excessive melting (red dotted rectangle), good melting (green dotted rectangle) and weak sintering (blue dotted rectangle). Typically, the width of the single line narrows as the laser power decreases and the scanning speed increases. Figure 9a clearly illustrates that the majority of the area displays a consistent and uninterrupted path, which provides the basis for subsequent bulk sample forming.

#### 3.3.2. Bulk Samples

The forming parameters of bulk samples depend on the process window of good melting in single-track samples. The hatch distance is set according to the melt pool overlap rate of 60%. The hatch distance is calculated using the following Formula (2). *Hr* represents the melt pool overlap rate, and *w* represents the width of the melt pool.

(2) h=(1−Hr)×w

**Figure 10:**
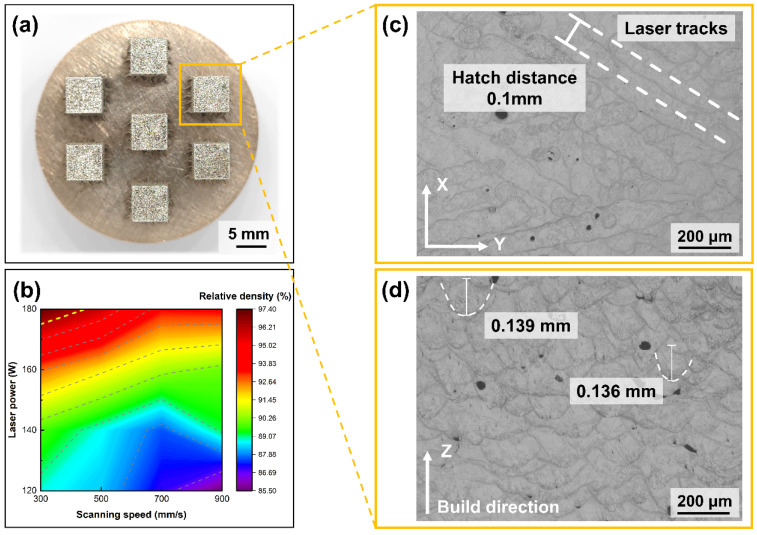
S800 Ag bulk sample, melt channel and melt pool images, (**a**) bulk sample; (**b**) relative density depends on laser power and scanning speed (**c**) melt pool morphology in XY plane; (**d**) melt pool morphology in Z plane.

As shown in Figure 10, dense LPBF sample have been successfully fabricated using the PREP S800 Ag powder. The scanning trajectory can be observed in the XY plane of S800 Ag by etching, which is consistent with the set scanning strategy and hatch distance. The overlapping melt pool in the Z plane (build direction) exhibits a “fish scale” characteristic. During LPBF, the high energy in the central region of the laser beam leads to the maximum depth of the melt pool, with the energy decreasing from the central region to the edges, thereby forming an arcuate “fish scale” cross-section [45]. As a new layer of powder is deposited on the top of the part, the laser melts the powder, causing the previously layer to be remelted. Variations in melt pool depth and shape lead to irregular wavy-shaped tracks.

The relative density of S800 Ag depends on the laser power and scanning speed shown in Figure 10b, which shows that the densities of the components increase with increasing laser power and decreasing scanning speed. The highest relative density (97.36%) was obtained using a laser power of 180 W and a scanning speed of 300 mm/s. It was proven that the S800 Ag powder prepared in this study for LPBF is completely feasible and can be used to fabricate LPBF parts with high density.

### 3.4. Microstructure and Defects

**Figure 11:**
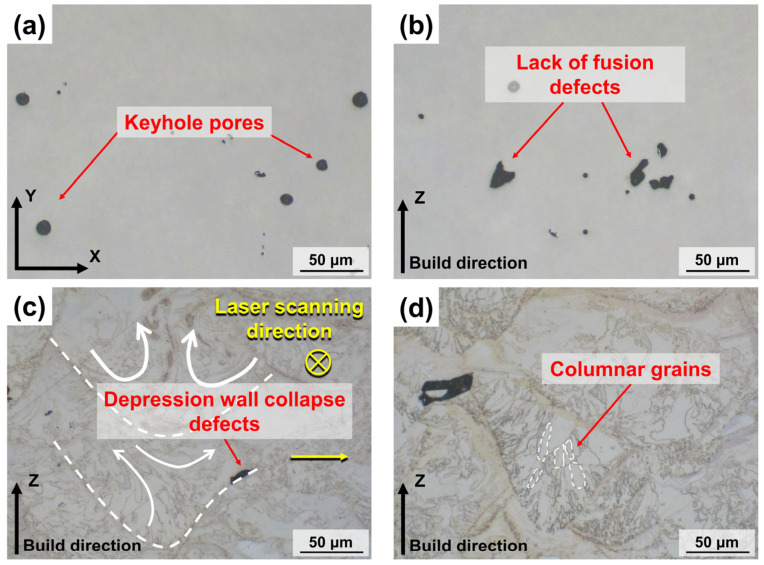
Internal defects and microstructure of S800 Ag LPBF samples; (**a**) keyhole pore defects; (**b**) lack of fusion defects; (**c**) depression wall collapse defects; (**d**) columnar grains growing in the direction of the thermal gradient.

Optical microscope images and the microstructure of the LPBF S800 Ag bulk samples are shown in Figure 11. Several internal defects can be found, which led to a decrease of relative density of the LPBF S800 Ag. In this study, the type of defect is indicated by roundness (Formula (1)). A roundness value of 0.8 or higher is classified as a keyhole pore defect, between 0.6 and 0.8 is classified as a lack of fusion defect and below 0.6 is classified as a depression wall collapse defect.

Keyhole pore defects can be found in Figure 11a. The keyhole pores are caused by the destabilisation of the melt pool due to the laser energy set up not being perfectly matched to the metal powder [46]. The formation of keyhole in the melt pool is a result of the metal vapour recoil pressure. As the metal vapour recoil pressure decreases and the surface tension of the metal liquid increases, the keyholes collapse. This collapse leads to the generation of pores at the bottom of the melt pool. However, these pores are unable to escape due to the rapid solidification of the melt pool, resulting in the formation of internal pores [47,48]. Lack of fusion defects can be observed in Figure 11b, which are caused by an insufficient laser energy input. The scanning tracks formed by low laser energy cannot form a good combination with the building layers, resulting in the formation of lack of fusion defects [49].

The formation of depression wall collapse defects at the edge of the melt pool is observed in Figure 11c. The molten metal first flows towards the outer edge of the melt pool under the action of the Marangoni flow and then returns to the centre of the melt pool. This movement causes the collapse of the melt pool, leading to the formation of pores at the front of the melt pool depression wall [50]. Many elongated columnar grains were observed in the Z-plane (Figure 11d), and the grains grew towards the centre of the melt pool along the build direction, which was due to the preferential growth of columnar grains along the direction of the thermal gradient during the gradual cooling [51].

### 3.5. Thermal Conductivity and Vickers Hardness

**Figure 12:**
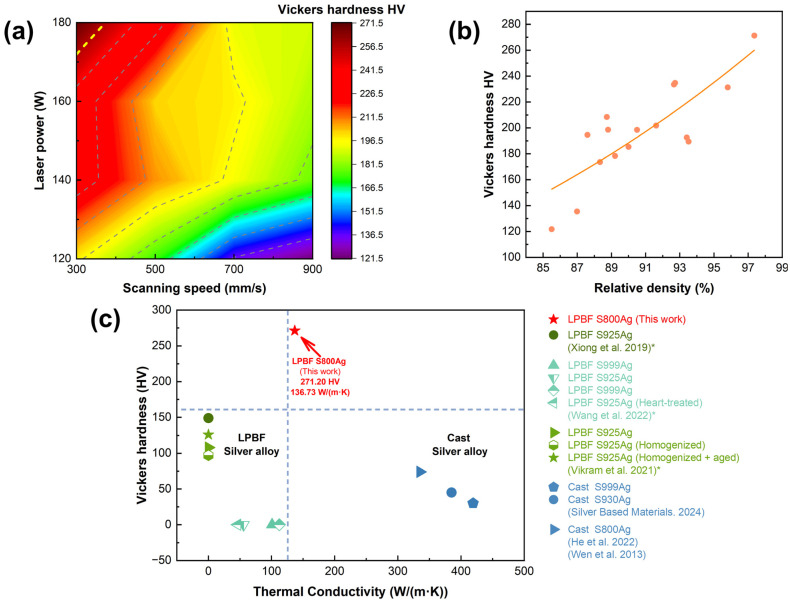
(**a**) Vickers hardness depend on laser power and scanning speed, (**b**) Vickers hardness depends on relative density; (**c**) Hardness and thermal conductivity of silver alloys in this and previous studies, *: Xiong et al. and Vikram et al. did not conduct research on thermal conductivity; Wang et al. did not conduct research on Vickers hardness [20,21,25,44,52,53].

The Vickers hardness of the same material is mainly affected by the macroscopic internal defects and microscopic grain size of the sample. Figure 12a shows the relationship between process parameters and Vickers hardness. The LPBF S800 Ag Vickers hardness decreases with decreasing laser power and increasing scanning speed. According to Figure 12b, there is a clear positive relationship between Vickers hardness and relative density. The sample with the highest density (97.36%) exhibited the highest Vickers hardness (271.20 HV). This suggests that the primary factor influencing hardness is the presence of macroscopic internal defects. Specifically, the internal pores are susceptible to collapsing when subjected to external forces, leading to a decrease in Vickers hardness. The disparity in Vickers hardness among samples with similar relative density can be attributed to variations in the size and morphology of the internal grains.

Figure 12c shows a comparison of the Vickers hardness and thermal conductivity of LPBF S800 Ag in this study with other silver alloys investigated in previous studies. The LPBF S800 Ag samples fabricated in this work exhibits superior Vickers hardness and thermal conductivity properties compare to the S925 Ag and S925 Ag LPBF samples in previous studies. However, the thermal conductivity remains inferior to that of conventionally cast parts. The thermal conductivity of traditionally cast S800 Ag is 334.9 W/(m·K) [52], whereas the LPBF printed S800 Ag exhibits a thermal conductivity of 136.73 W/(m·K). The primary mode of heat conduction in metal is through the movement of electrons. The presence of numerous small, closed circular holes and irregular pores on both the interior and surface of the LPBF samples lead to a lower relative density (97.36%) compared to conventionally casted samples. These features hinder the movement of electrons, thereby reducing the overall thermal conductivity. The rapid melting and cooling rate during LPBF induced the grain refinement and increased residual stresses [54,55]. As a result, the Vickers hardness of LPBF S800 Ag, which is 3.66 times higher than that of mould casting, significantly enhances material strength and abrasion resistance, the application range of silver alloys will be further expanded.

## 4. Conclusions

The hardness of the S800 Ag electrode rod for PREP was improved using continuous casting. The employment of continuous casting technology effectively solved the problems of inadequate hardness and defects frequently observed in traditional silver mould casting, as well as the issue of bending during high-speed rotation. S800 Ag powders with good sphericity and high sphericity ratio were successfully prepared by “CC + PREP”. Furthermore, dense S800 Ag was successfully fabricated by LPBF.

1. The hardness of the silver alloy electrode rod was effectively enhanced through the continuous casting process. The fine dendrites were induced due to the outer edge of the rods undergoing rapid cooling using the water-cooled crystalliser. The hardness was enhanced by the reinforcement of these fine crystals, resulting in a 30.30% increase in hardness compared to S800 Ag prepared by traditional mould casting.
2. The S800 Ag electrode rod with enhanced hardness enabled stable rotation with the speed up to 25,000–37,000 rpm. The silver alloy powders prepared by “CC + PREP”, which has a small particle size (15–60 μm), satisfy the requirements for LPBF fabrication. Furthermore, S800 Ag powder showed a 5.56% increase in average sphericity (0.98) and a 36.28% increase in sphericity ratio (97.67%) compared to the GA silver alloy powders.
3. The LPBF process rapidly cooled the material, resulting in a crystalline reinforcement that provided S800 Ag with a Vickers hardness (271.20 HV) 3.66 times higher than mould casting. The strength was significantly improved, hence facilitating the development of silver alloy components with high strength and complex structures.

The present study proposes the “CC + PREP” approach as a solution to the issue of low speed of rotation during PREP due to the low hardness of silver alloy electric rods using mould casting. Further research will be conducted to explore in typical silver alloys such as S925 Ag and S999 Ag. The optimisation of the LPBF parameters will be applied using the high power of multi lasers to improve the density for the application of large silverware and electric device.

## Figures and Tables

**Table 1:** Parameters of silver alloy powders prepared by PREP used in this paper.

Plasma Gas	Rotational Speed (rpm)	DC Current (A)	Feeding Rate (mm/s)
Ar	25,000–37,000	500–700	3.5–4.5

**Table 2:** Data of S800 Ag powders prepared by PREP.

	Particle Size Distributions	Apparent Density	Tap Density
S800 Ag	D_10_ = 23.08 μm D_50_ = 34.42 μm D_90_ = 51.04 μm	5.92	6.20

## Data Availability

Data are contained within the article.
